# Supplementary material for: MetaQTL: a package of new computational methods for the meta-analysis of QTL mapping experiments
Source: BMC Bioinformatics. 2007 Feb 8;8:49. doi: 10.1186/1471-2105-8-49 (PMC1808479; doi:10.1186/1471-2105-8-49)
Supplement: Additional File 7 — MetaQTL Package : jar file and tutorial. This Zip archive contains both the MetaQTL JAR file and the files of the tutorial. [file 1471-2105-8-49-S7.zip › org.inra.metaqtl/doc/org/metaqtl/factory/MetaMapSummaryFactory.html]

MetaMapSummaryFactory


|  |  |  |
| --- | --- | --- |
| **Overview** \| **Package** \| **Class** \| **Use** \| **Tree** \| **Deprecated** \| **Index** \| **Help** | |  |
| **PREV CLASS**   **NEXT CLASS** | **FRAMES**    **NO FRAMES**     **All Classes** |  |
| SUMMARY: NESTED \| FIELD \| CONSTR \| METHOD | DETAIL: FIELD \| CONSTR \| METHOD |  |


---


## org.metaqtl.factory Class MetaMapSummaryFactory

```
java.lang.Object
  org.metaqtl.factory.MetaMapSummaryFactory
```

---

``` public class MetaMapSummaryFactory extends java.lang.Object ```

A factory to write out the information on a `MetaMap`.

---

**Constructor Summary**

`MetaMapSummaryFactory()`


| **Method Summary** | |
| --- | --- |
| `static void` | `write(MetaMap metaMap, java.io.OutputStream stream)`             This methods writes out the summary for a consensus genetic map into a given output stream `stream`. |

**Methods inherited from class java.lang.Object**

`clone, equals, finalize, getClass, hashCode, notify, notifyAll, toString, wait, wait, wait`

**Constructor Detail**

### MetaMapSummaryFactory

```
public MetaMapSummaryFactory()
```


**Method Detail**

### write

```
public static void write(MetaMap metaMap,
                         java.io.OutputStream stream)
                  throws java.io.IOException
```

:   This methods writes out the summary for a consensus genetic map into
    a given output stream `stream`. The user is reponsible for
    closing the stream.
    The format of the output is as follows :

    - CH the name of the chromosome.
    - NM the number of markers on the chromosome.
    - GF the goodness-of-fit.
    - PV the p-value.
    - DF the degress of freedom.
    - SR the standard residuals for the marker intervals of the
      imput maps.

    :   **Parameters:**: `metaMap` - a consensus genetic map.: `stream` - an output stream. **Throws:**: `java.io.IOException`


---


|  |  |  |
| --- | --- | --- |
| **Overview** \| **Package** \| **Class** \| **Use** \| **Tree** \| **Deprecated** \| **Index** \| **Help** | |  |
| **PREV CLASS**   **NEXT CLASS** | **FRAMES**    **NO FRAMES**     **All Classes** |  |
| SUMMARY: NESTED \| FIELD \| CONSTR \| METHOD | DETAIL: FIELD \| CONSTR \| METHOD |  |


---
